# LncRNA LINC02257: A Potential Biomarker for Diagnosis and Prognosis of Colorectal Cancer

**DOI:** 10.1155/2022/4330630

**Published:** 2022-09-10

**Authors:** Mei Chen, Changbing Li, Qinghua Luo, Anhui Tan

**Affiliations:** Department of Anorectal, The National Hospital Of Enshi Autonomous Prefecture, Enshi, Hubei, China

## Abstract

Colorectal cancer (CRC) is the third most common cancer and the second leading cause of cancer mortality worldwide. However, efficient markers for CRC diagnosis are limited. Accumulating evidence reveals that long noncoding RNAs (lncRNAs) are related to the genesis and developments of many tumors. In this study, we aimed to explore the diagnostic and prognostic value of LINC02257 in CRC patients. TCGA datasets were utilized to examine LINC02257 expression in a variety of human malignancies. The Kaplan–Meier method analysis was then used to study the link between LINC02257 expression and patient prognosis. Multivariate assays were applied for the determination of the associations of the variables and patients' survivals. RT-PCR was used to examine the level of LINC02257 expression in 14 pairs of clinical CRC tissues as well as many distinct CRC cell lines. CCK-8 assay was used to assess cell proliferation. We found that the expression of LINC02257 exhibited variable patterns of upregulation or downregulation in the various forms of cancer. In CRC, LINC02257 expression was distinctly increased in CRC specimens compared with normal specimens. The results of ROC curves revealed that the AUC was 0.886 (0.862 to 0.909, 95% CI, *p* < 0.001) in a comparison between CRC specimens and matched normal specimens. Survival studies revealed that high LINC02257 expression was associated with shorter overall survival and disease specific survival. More importantly, multivariate assays confirmed that high expression of LINC02257 was an independent prognostic factor for CRC patients. The results of RT-PCR indicated that LINC02257 expression was distinctly overexpressed in both CRC specimens and cell lines. Functionally, silence of LINC02257 distinctly suppressed the proliferation of CRC cells. In conclusion, our research showed that LINC02257 is an intriguing candidate as a diagnostic and prognostic indicator for patients diagnosed with CRC.

## 1. Introduction

Colorectal cancer (CRC) belongs to the globally commonest malignancies, with almost 1.35 million new cases and 0.8 million deaths annually [1]. Recent years have shown a trend toward an increased occurrence of this condition, which is consistent with the tremendous shift in dietary composition and lifestyle that has occurred all across the world [2]. The widespread use of surgical resection, which may or may not be accompanied by adjuvant chemotherapy and radiation therapy depending on the clinical stages of tumors, has led to an improvement in the clinical outcome for a significant number of patients with CRC [3, 4]. On the other hand, it has been discovered that some patients who undergo early surgery suffer from distant metastases, in particular liver metastases, which may ultimately result in the treatment not working [5, 6]. Therefore, it is of the utmost importance to locate sensitive diagnostic and prognostic biomarkers in order to establish the most effective therapy methods for patients suffering from CRC.

The great advancements that have been made in genome and transcriptome sequencing in recent years have allowed for the discovery of a large number of genes that do not code for proteins [7]. These genes make up approximately 75% of the genome. RNAs that are longer than 200 nucleotides and are transcribed from genes that do not code for proteins are referred to as long noncoding RNAs (lncRNAs) [8]. Despite the fact that lncRNAs are unable to code for proteins, which limits their biological function in the growth of cells, there is growing evidence that many lncRNAs have the potential to affect genetic control, epigenetic regulation, and post-transcriptional regulation [9, 10]. Recent research has shown that a number of lncRNAs are aberrantly expressed in a variety of tumors. These lncRNAs have the potential to play either an oncogenic or an antioncogenic role in the oncogenesis and progression of different types of tumors by participating in a sequence of cellular progressions, such as cellular growth and distant metastasis [11, 12]. In addition, an increasing number of studies suggested the value of using lncRNAs as potential diagnostic and prognostic markers for patients suffering from various forms of cancer, including CRC. Despite this, there were still a significant number of lncRNAs that needed to be identified clinically.

Long intergenic nonprotein coding RNA 2257 (LINC02257) is a newly identified lncRNA which is located on 1q41. To date, the study of LINC02257 on tumors was rarely reported. Its expression and prognostic value were just reported in kidney renal clear cell carcinoma and CRC [13, 14]. However, the studies are limited. The purpose of this study was to investigate the expression pattern, clinical relevance, and potential of LINC02257 in patients with CRC.

## 2. Patients and Methods

### 2.1. Data Collection

RNA-seq profiles of 480 cases of colorectal cancer and 41 samples of normal tissue were collected using TCGA database (https://portal.gdc.cancer). In addition to that, we derived demographic information from these samples.

#### 2.1.1. Patients and Tissue Samples

CRC specimens and adjacent normal specimens, which were histopathologically confirmed by two experienced pathologists, were collected from 14 CRC patients who underwent surgery at the First Affiliated Hospital of The National Hospital of Enshi Autonomous Prefecture from July 2020 to June 2021. All cases received no preoperative adjuvant therapies such as radiotherapy and chemotherapy. The follow-up period for all cases was five years. All collected specimens (CRC tissues and normal specimens) were snap-frozen in liquid nitrogen and stored at −80°C immediately after resection for further RT-PCR assays. Written informed consent for the application of biological specimens was obtained from each patient involved in the study, and the ethics committee of our hospital approved this study.

#### 2.1.2. Cell Lines

Five CRC cell lines (HT29, SW480, HCT15, HCT116, and DLD1) and one normal colonic epithelial cell line (FHC) were obtained from the American Type Culture Collection. The standard culture media for all of the cell lines was Dulbecco's modified Eagle's medium (DMEM; Gibco BRL, Grand Island, New York, United States), which contained 10 percent fetal bovine serum (FBS; Gibco BRL). The lipofectamine RNAiMAX reagent from Thermo Fisher Scientific, Waltham, Massachusetts, was used to transfect cells with small interfering RNAs (siRNAs), as outlined in the protocol provided by the manufacturer. GenePharma was responsible for the design and synthesis of both the LINC02257 siRNA (si-LINC02257) and the negative control siRNA (si-NC).

#### 2.1.3. The Real-Time Reverse Transcription Polymerase Chain Reaction (RT-PCR)

Total RNA from all samples of 14 CRC patients was isolated using the TRIzol reagent (Invitrogen, Hangzhou, Zhejiang, China). A reaction mixture containing 1 *μ*g of total RNA was reversely transcribed to cDNA using Synthesis SuperMix (Transgene Biotek, Ltd., Hyderabad, India). Real-time PCR detection of genes was carried out by the use of the SYBR Green Master Mix (Biosystems, Xunwu, Nanjing, China). All reactions were run in triplicate. The LINC02257 level was calculated with the 2^−ΔΔCt^ methods, which was normalized to GAPDH. The expressions of LINC02257 and GAPDH were relative to the fold change of the matched normal specimens, which were defined as 1.0. The primer sequences were presented as follows: LINC02257 5′-CTCTAGCCTCTGGCATCACAG-3′ (forward) and 5′- CTCCACTAGGCTCGCCACG′(reverse), and GAPDH 5′- GGTGAAGGTCGGAGT CAACG-3′ and 5′- CAAAGTTGTCATGGATGHACC -3′.

#### 2.1.4. Cell Counting Kit-8 (CCK-8)

Both DLD1 and SW480 cells were grown in 96-well plates at a density of 4 × 10^4^ cells per well for a period of 24 hours. After that, 10 *μ*l of a solution from a cell counting kit-8 (CCK-8) manufactured by Dojindo Laboratories, Inc (Pudong, Shanghai, China) was added to the medium, and the cells were then incubated for 2 hours at 37°C in an atmosphere containing 5% CO_2_. With the use of a Spectrafluor microreader plate, the value of the optical density was determined at a wavelength of 450 nm (Molecular Devices, LLC). These trials were carried out three times in all.

### 2.2. Statistical Analysis

All statistical data were analyzed by SPSS 18.0 software (SPSS, Chicago, IL, USA). Statistical analyses were carried out by the use of either an analysis of variance (ANOVA) or Student's *t*-test. Receiver operating characteristic (ROC) curves were established to examine the possible clinical value of LINC02257 expression for CRC diagnosis. The Kaplan–Meier method with the log-rank test for comparisons was used to calculate overall survival (OS) and disease-specific survival (DSS) rates. Univariate and multivariate assays were applied for the determination of the associations of the variables and patients' survivals. A *p* value <0.05 was considered to be statistically significant.

## 3. Results

### 3.1. Pan-Cancer Analysis of LINC02257 Expression Levels

**Figure 1:**
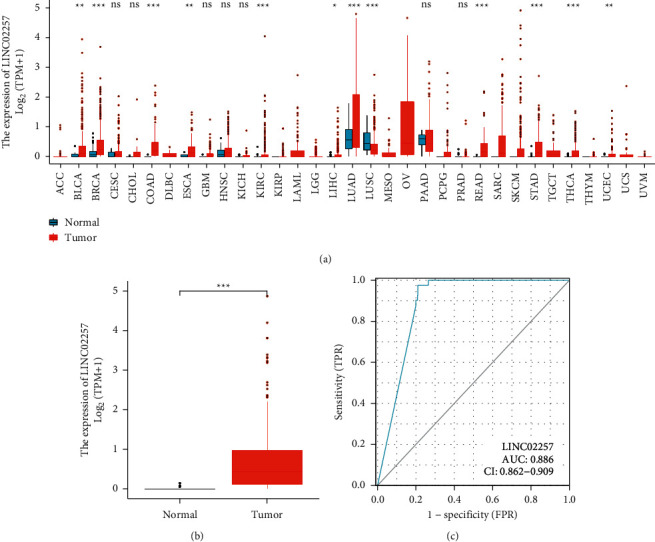
Expression of LINC02257 in TCGA cohorts. (a) Differential expressions of LINC02257 between non-tumor and tumor specimens in TCGA datasets. the *X* axis represents the expression of LINC02257 and the *X* axis represents the names of tumors. (b) LINC02257 was overexpressed in CRC specimens compared with non-tumor specimens. (c) Receiver operator characteristic curve analysis of LINC02257.

**Figure 2:**
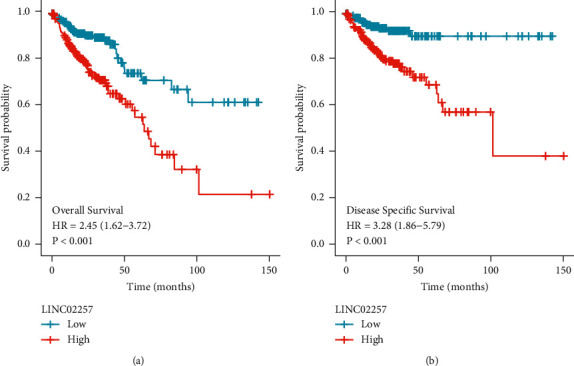
Kaplan-meier methods estimating the overall survivals and disease specific survivals according to the expression of LINC02257 in patients with CRC.

For the purpose of investigating the role that LINC02257 plays in cancer, the expression levels of LINC02257 were evaluated across a wide spectrum of cancers using TCGA datasets. According to the findings, the expression of LINC02257 exhibited variable patterns of upregulation or downregulation in the various forms of cancer (Figure 1(a)). We were able to discover that the expressions of LINC02257 were distinctly increased in the majority of different types of cancers. These findings provide evidence of the inherent changes in the expression of LINC02257 that exist between the various types of tumors. Detailed assessments of the expression of LINC02257 were taken into consideration for further study. Importantly, our group observed that the expressions of LINC02257 were distinctly upregulated in CRC specimens compared with normal specimens (Figure 1(b)). The significant upregulation of LINC02257 in CRC patients encouraged us to further explore its diagnostic value for CRC patients. As shown in Figure 2, the results of ROC curves revealed that the AUC was 0.886 (0.862 to 0.909, 95% CI, *p* < 0.001) in a comparison between CRC specimens and matched normal specimens (Figure 1(c)).

### 3.2. Association of LINC02257 Expression with Clinicopathological Features of CRC Patients

To explore the clinical effect of LINC02257 on the progression of CRC patients, 478 CRC samples were divided into two subgroups high-group: *n* = 239 and low-group: *n* = 239 according to the median ratio of relative LINC02257 expressions. The chi-square test suggested that high LINC02257 expression in 239 CRC patients was distinctly associated with the pathologic stage (*p*=0.003) (Table 1). However, there were no significant associations between LINC02257 expressions and other clinical features.

### 3.3. Association between LINC02257 Expression and Patient Survival

Then, we explored whether there are any associations between dysregulated expression of LINC02257 and clinical survivals of CRC patients. First, we analyzed the survival data from a cohort (478 CRC patients) from TCGA datasets, finding that patients with high LINC02257 expressions had shorter OS (*p* < 0.001, Figure 2(a)) and DSS (*p* < 0.001, Figure 2(b)) time than those with low LINC02257 expressions. To further determine the prognostic values of LINC02257 expression in CRC patients, we performed univariate and multivariate assays which suggested that high expression of LINC02257 was an independent prognostic factor for both OS (HR = 2.13, 95% CI: 1.395–3.280; *p* < 0.001, Table 2) and DSS (HR = 2.574, 95% CI: 1.450–4.569; *p* < 0.001) (Table 3).

### 3.4. The Upregulation of LINC02257 Expression in CRC and Its Oncogenic Roles

**Figure 3:**
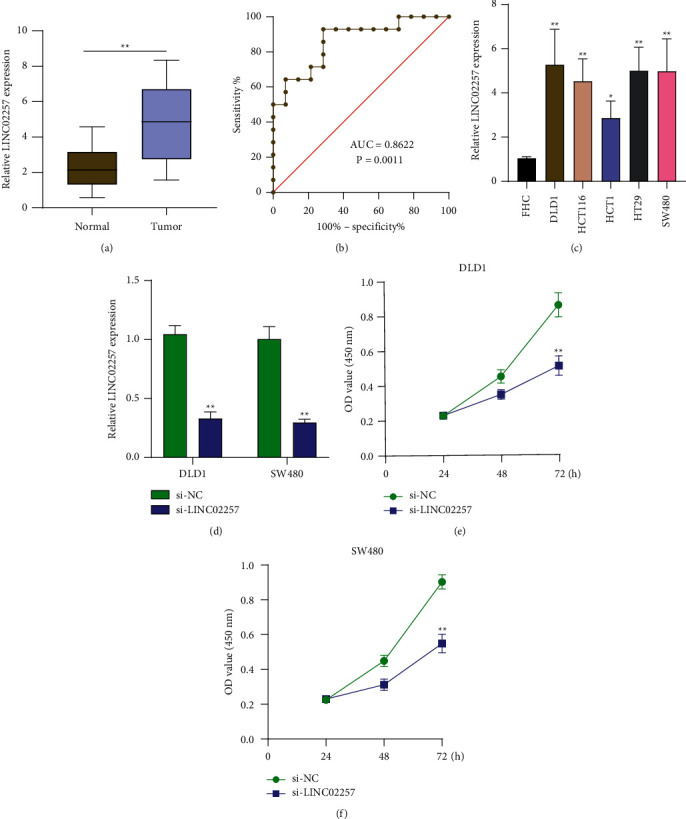
The expression of LINC02257 in our cohort and its functional roles. (a) The LINC02257 relative expression levels were determined by qRT-PCR in 14 pairs of CRC tissues and the adjacent non-tumor specimens. (b) The ROC curve analysis for discriminative ability between CRC specimens and normal tissues. (c) LINC02257 expressions were increased in CRC cell lines compared to normal FHC cells. (d) RT-PCR analysis of LINC02257 expression levels following DLD1 and SW480 cell treatment with si-LINC02257.

Then, we carried out RT-PCR to examine the levels of LINC02257 in 14 pairs of CRC specimens and nontumor specimens. As shown in Figure 3(a), we discovered that the levels of LINC02257 was noticeably increased in CRC tissues in comparison to nontumor specimens. Moreover, the results of ROC assays revealed that the AUC was 0.8622 (*p* < 0.001) in a comparison between CRC specimens and matched normal specimens (Figure 3(b)). Next, LINC02257 expression was detected by qRT-PCR in 5 human colorectal cancer cell lines (Figure 3(c)). Notably, all the cell lines expressed higher levels of LINC02257 versus the FHC, but DLD1 and SW480 cells expressed relatively higher levels of LINC02257 compared with other three cells. Therefore, we chose DLD1 and SW480 for further studies. For the silencing assays, cells were treated with LINC02257 siRNA or scramble siRNA controls. LINC02257 levels were significantly reduced in DLD1 and SW480 after siRNA treatments, compared to controls (Figure 3(d)). The results of further CCK-8 experiments demonstrated that silence of LINC02257 markedly suppressed the growth of DLD1 and SW480 cells (Figures 3(e) and 3(f)).

## 4. Discussion

The most common cause of death from colorectal cancer is the progression of metastasis, and the liver is the primary organ in which metastatic colonization can be found in more than 65 percent of CRC patients [15]. It is highly important for the reduction in the number of occurrences of metastatic illness to have both an early diagnosis and a treatment plan that is optimized based on the possible prognosis of CRC patients [16, 17]. In recent years, an increasing number of studies have reported that long noncoding RNAs, or lncRNAs, play an important role in the progression of tumors. Furthermore, their abnormal expressions in the cancer specimens and blood of patients suggested that lncRNAs may have the potential to be used as novel biomarkers [18, 19]. Several long noncoding RNAs have been proven to have a positive association with the long-term survivals of colorectal cancer patients and to exhibit clinical significance in differentiating cancer specimens from nontumor samples.

In this research, our group observed that the expressions of LINC02257 exhibited an increased trend in many types of tumors, which suggested its oncogenic roles in tumor progression. However, there may be some abnormal phenomena. Some lncRNAs are highly expressed in some tumors, but their overexpression deficiency can inhibit tumor proliferation and metastasis, which may be due to the complex mechanisms involved in tumor progression [20, 21]. For LINC02257, we have looked up a lot of literature. Only some studies reported that LINC02257 was highly expressed in some tumors, such as kidney renal clear cell carcinoma and CRC [13, 14]. However, there was no in vivo or in vitro experimental study on its function. Here, we found that LINC02257 expressions were distinctly increased in CRC, which was consistent with previous findings. In addition, we confirmed that this lncRNA has certain diagnostic significance via ROC assays. Clinical assays confirmed that LINC02257 was an independent poor prognostic factor for both OS and DSS. On the other hand, we firstly studied the potential function of LINC02257 in CRC progression, finding that silence of LINC02257 exhibited a suppressor effect in the proliferation of CRC cells, suggesting it as an oncogene in CRC progression. Our findings first reported that this lncRNA has a carcinogenic effect.

However, there are still some shortcomings in this study. First, it is a retrospective research, and as such, it suffers from the inherent selection bias and reporting bias that plague all retrospective studies. Due to the limited number of patients who participated in our study, additional research including a substantial number of patients is necessary to validate the results of our investigation. Second, our data are restricted to overall patient survival, and it will be of interest to analyze the association of LINC02257 expression with cancer recurrences. Third, in this article, the functional mechanisms are not investigated in great detail. Therefore, to compensate for these shortcomings, additional research ought to be carried out.

## 5. Conclusion

We identified a novel CRC-related lncRNA, LINC02257, which could be used as a potential marker for CRC patients. To further show the prognostic and diagnostic significance of LINC02257 in patients with CRC, additional in-depth investigations are required.

## Figures and Tables

**Table 1:** Association between LINC02257 and clinicopathological parameters of CRC.

Characteristic	Low expression of LINC02257	High expression of LINC02257	*p*
*n*	239	239	

Gender, *n* (%)			0.927
Female	112 (23.4%)	114 (23.8%)	
Male	127 (26.6%)	125 (26.2%)	

Age, *n* (%)			0.780
<=65	99 (20.7%)	95 (19.9%)	
>65	140 (29.3%)	144 (30.1%)	

Pathologic stage, *n* (%)			0.003
Stage I	53 (11.3%)	28 (6%)	
Stage II	95 (20.3%)	92 (19.7%)	
Stage III	60 (12.8%)	73 (15.6%)	
Stage IV	24 (5.1%)	42 (9%)	

Age, median (IQR)	68 (59.5, 76.5)	69 (58, 78)	0.550

**Table 2:** Univariate and multivariate analysis of overall survival in CRC patients.

Characteristics	Total (*N*)	Univariate analysis		Multivariate analysis	
		Hazard ratio (95% CI)	*p* value	Hazard ratio (95% CI)	*p* value
Age	477				
<=65	194	Reference			
>65	283	1.610 (1.052–2.463)	**0.028**	2.114 (1.354–3.299)	**<0.001**

Gender	477				
Female	226	Reference			
Male	251	1.101 (0.746–1.625)	0.627		

Pathologic stage	466				
Stage I & stage II	267	Reference			
Stage III & stage IV	199	2.947 (1.942–4.471)	**<0.001**	3.114 (2.033–4.768)	**<0.001**

LINC02257	477				
Low	238	Reference			
High	239	2.451 (1.617–3.716)	**<0.001**	2.139 (1.395–3.280)	**<0.001**

**Table 3:** Univariate and multivariate analysis of disease specific survival in CRC patients.

Characteristics	Total (*N*)	Univariate analysis		Multivariate analysis	
		Hazard ratio (95% CI)	*p* value	Hazard ratio (95% CI)	*p* value
Age	461				
<=65	191	Reference			
>65	270	1.165 (0.702–1.933)	0.555		

Gender	461				
Female	220	Reference			
Male	241	1.142 (0.697–1.871)	0.599		

Pathologic stage	451				
Stage I & stage II	259	Reference			
Stage III & stage IV	192	6.085 (3.235–11.447)	**<0.001**	5.533 (2.934–10.432)	**<0.001**

LINC02257	461				
Low	230	Reference			
High	231	3.280 (1.860–5.785)	**<0.001**	2.574 (1.450–4.569)	**0.001**

## Data Availability

The data used to support the findings of this study are available from the corresponding author upon request.
